# Meeting in Dublin

**Published:** 1917-02-03

**Authors:** 


### THE ROYAL BRITISH COLLEGE OF NURSING.

### Meeting in Dublin.

A MEETING to discuss the College of Nursing was held on Saturday, January 27, at 5 P.M., in the College of Physicians, Dublin, Dr. Joseph O'Carroll, M.D., President of the Royal College of Physicians, in the chair, and the audience was large and representative.

The Chairman stated that the object of the meeting was that those present might learn about the Royal British College of Nursing and to discuss whether it would be advisable for Irish nurses to join the organisation or not, but it was not intended that any resolutions should be passed or that they should arrive at any conclusion.

Miss Cox-Davies, R.R.C., Matron Royal Free Hospital, then addressed the meeting, and said that Miss Haughton, Matron of Guy's, was the speaker who should be addressing the meeting, but owing to her illness she (Miss Cox-Davies) came instead. Though it was her first visit to Ireland she knew a good deal of Irish nurses and had had many of them in responsible posts under her. Miss Cox-Davies went on to say that she had been a keen Registrationist all through her nursing career, and therefore took a great interest in the College of Nursing, as she believed that it was through it nurses would get State Registration. She was sure there were many in the room who had not yet made up their minds as to whether or not this College would be of good service to the profession; but she asked all those present to work it out with an unbiassed mind, remembering that a great change had taken place since 1914, and to think of the educational value of such a College. She hoped that the result of the meeting would be that she and Miss Rundle would carry with them the good wishes and co-operation of the Irish nurses.

It was impossible to speak of the founding of the College without thinking of the leaders in the earlier movement for State Registration. Those who stood out remarkably were Miss Isla Stewart, Matron of St. Bartholomew's, who went to her rest before seeing the fruits of her work, and Mrs. Bedford Fenwick. It was hardly necessary to speak of the work of Mrs. Bedford Fenwick to Irish nurses, and of all nurses owed to her in the movement for State Registration, but she was sorry to say some of these leaders did not see quite eye to eye with those connected with the present scheme. Whenever or however they got registration they would always owe a great debt of gratitude to those early leaders.

Miss Cox-Davies then paid a tribute to the V.A.D.s, and said it would have been impossible for the trained nurses to cope with the large numbers of sick and wounded but for the whole-hearted way in which these girls threw themselves body and soul into the work, and it was owing to these V.A.D.s (many of whom had left sheltered homes to do work which was hard enough to the women who had been trained to it) that the trained women had been able to nurse the wounded as they had done, and some of the greatest sacrifices made during the war are being made by the parents of V.A.D.s who allowed their daughters to take up hospital work.

The danger after the war to trained nurses from the V.A.D.s she believed to be non-existent, as large numbers of V.A.D.s would return to civil life, and the others would recognise that they should take out a full training. Mr. Stanley thought that the scheme of the College was the one solution of the difficulty, and that some great educational body should be started which would gain the support also of those who were against the previous Bill, and that a voluntary Register should be kept by the College, which should be a State Register. Mr. Stanley set to work to find out the views of the nursing profession, and when he found there was a demand for State Registration, he put State Registration as the first thing the College should strive for. One of the greatest difficulties at first was that the training schools did not forward the movement, and no Bill for State Registration would stand a good chance of being carried through the House of Commons which did not carry with it the good will and support of the training schools.

At a meeting held in London on January 18, 1917, and after careful deliberation, the College of Nursing, Ltd., amalgamated with the British Nursing Association, and the name of the College became the Royal British College of Nursing. The word "Ltd." has been dropped, and the reason why it was ever used was because Mr. Stanley had made up his mind that Registration was the one thing to be fought for, and that incorporating the College under the Board of Trade would be the shortest way to get it. Unity in the nursing profession is the most important point towards getting registration. The obtaining of the Royal Charter is owing to Mrs. Bedford Fenwick's untiring work, and the College hopes the time will come when it will be able to work with her instead of leaving her behind. Miss Cox-Davies went on to say that the College was established whether the nurses wanted it or not, and would continue to help the nursing profession by its Register, which is growing by leaps and bounds in the hope that it would become a State Register. When the Bill goes to Parliament depends largely on when it receives the support of the whole of the nursing profession. Scotland has already formed its local boards, and what is now needed is for Ireland to decide whether she wants to be included in the College.

There were problems in Ireland as well as in Scotland and England which needed solving, and it would be better for Ireland to have its own local board sitting on the Council of the College from the start. Miss Cox-Davies asked the nurses as well as the matrons and superintendents who were present to decide whether they did or did not wish to be represented on the Council of the College of Nursing. Miss Cox-Davies said that she and Miss Rundle had come over from England for the purpose of answering questions, and that they had been greatly helped by paragraphs in Irish newspapers, of which they had made careful notes, and that the one way in which Irish nurses could help was by building up the Register and so strengthening the hands of those going to Parliament with the Bill. Some Irish nurses had registered and others were waiting the formation of an Irish Board. Miss Cox-Davies then said, "I ask you to help forward the movement which is to bring the crown to all the work which has been done by those workers in all the years gone by."

Miss Rundle, R.R.C., the Secretary of the College of Nursing, then addressed the meeting and explained that the Council realised that there were questions in

(*Continued on page 374.*)

### The Royal British College of Nursing: Meeting in Dublin (*continued from p.* 372).

Ireland and Scotland which English nurses could not solve, so the representatives of the local boards would come to London and help to settle these questions. Scotland had already formed its local board and had its representatives on the Council. There were still six places left unfilled, and she hoped those six would be filled by Irish nurses. If not, the Privy Council might ask, "Where are the Irish nurses?" They had numbers of applications from Irish nurses for membership, and these nurses were still waiting a reply as to whether they were accepted or not. Miss Rundle could not yet tell them, as she was waiting for the formation of an Irish Board.

One of the difficulties was the difference in the training curriculum of England, Ireland, and Scotland. The aim of the College was not a uniform curriculum, but a uniform standard of training, as it was very hard to be fair to nurses applying for registration when the standard was different. They had refused hundreds and accepted thousands. Miss Rundle said she had seen it stated in the *Weekly Irish Times* that Irish nurses would have to go across to London to attend lectures and be examined. This was not true. Irish nurses would be educated in Ireland and examined in Ireland by examiners nominated by the Irish Board with the cooperation of the Council in London.

Then Miss Rundle spoke of finances. If representatives from Ireland were to go to London from time to time they would need money to pay their way. The Scottish Board was asked to state how much money they would need to pay the expenses of an office, a secretary, and the travelling expenses of their representatives. They told the Council how much they would want, and a cheque was drawn. The same thing would be done for the Irish Board. Scottish nurses now had their office in Edinburgh, where a number of nurses were dealing with registration forms sent to them from London.

Miss Rundle then appealed to the nurses' spirit of service, which she said was really the spirit of the profession. The nurses essentially served the patients and doctors, but not sufficiently each other. She asked them to think less of their own personal needs and more of the needs of the profession as a whole, and so build up for the United Kingdom a profession of which they would be truly proud, with some good education at the back of it.

The Chairman then invited any who desired to ask questions to do so, and started by asking, "How can Ireland come into a project which is named 'The British College of Nursing'?" The name of this kingdom is either "United Kingdom" or "Great Britain and Ireland," and he suggested that if the College chose to change its name to that of "Great Britain and Ireland," Irish nurses might come in. Another point that he raised was whether the College proposes to issue different diplomas for the separate branches of nursing. And, if so, what would prevent nurses having special diplomas from taking up work for which they were not qualified?

The Right Hon. Michael Cox rose in support of the suggestion of the Chairman that the College should incorporate Ireland in its name, saying that the medical profession were fond of the nurses and thought very highly of them, and he was pleased to hear that they need not attend in London to qualify for nursing diplomas.

Miss Cox-Davies, in reply, said with regard to the title of the College, "I am quite sure there was no intention to put on one side the individuality of Ireland, which we all wish to retain," and explained that its present name was due to the desire of the Royal British Nursing Association to retain its identity when the amalgamation was effected. She acknowledged the force of the point that the name ought to be changed to include Ireland, and thought the suggestion might be carried out with the assistance of Irish representatives. With reference to the granting of certificates in special subjects, no certificate would be granted unless the candidate had satisfied the Council that she had completed the full course of training and passed the portal exam.

The Chairman considered that the change of title should be a preliminary before an Irish Board was formed.

The meeting closed with votes of thanks to Miss Cox-Davies and Miss Rundle, the Chairman, and the Royal Colleges of Physicians and Surgeons. Very many questions were put and replied to, with which we hope to deal on another opportunity.

